# 2′-Fluoro-Pyrimidine-Modified RNA Aptamers Specific for Lipopolysaccharide Binding Protein (LBP)

**DOI:** 10.3390/ijms19123883

**Published:** 2018-12-05

**Authors:** Jasmin Aldag, Tina Persson, Roland K. Hartmann

**Affiliations:** 1 Jasmin Aldag, EUROIMMUN AG, Seekamp 31, D-23560 Lübeck, Germany; j.aldag@euroimmun.de; 2 Tina Persson, Passage2Pro AB, Östra Kristinelundsvägen 4B, SE-21748 Malmö, Sweden; tina@passage2pro.com; 3 Roland K. Hartmann, Philipps-Universität Marburg, Institut für Pharmazeutische Chemie, Marbacher Weg 6, D-35037 Marburg, Germany

**Keywords:** in vitro selection, aptamer, 2′-fluor-pyrimidine-modified, lipopolysaccaride binding protein (LBP), sepsis, systemic inflammatory response syndrome (SIRS)

## Abstract

Lipopolysaccaride binding protein (LBP), a glycosylated acute phase protein, plays an important role in the pathophysiology of sepsis. LBP binds with high affinity to the lipid part of bacterial lipopolysaccaride (LPS). Inhibition of the LPS-LBP interaction or blockage of LBP-mediated transfer of LPS monomers to CD14 may be therapeutical strategies to prevent septic shock. LBP is also of interest as a biomarker to identify septic patients at high risk for death, as LBP levels are elevated during early stages of severe sepsis. As a first step toward such potential applications, we isolated aptamers specific for murine LBP (mLBP) by in vitro selection from a library containing a 60-nucleotide randomized region. Modified RNA pools were transcribed in the presence of 2′-fluoro-modified pyrimidine nucleotides to stabilize transcripts against nuclease degradation. As verified for one aptamer experimentally, the selected aptamers adopt a “three-helix junction” architecture, presenting single-stranded 7-nt (5′-YGCTTCY) or 6-nt (5′-RTTTCY) consensus sequences in their core. The best binder (aptamer A011; *K*_d_ of 270 nM for binding to mLBP), characterized in more detail by structure probing and boundary analysis, was demonstrated to bind with high specificity to murine LBP.

## 1. Introduction

The lipid A component of lipopolysaccharide (LPS, endotoxin) represents the toxic agent of LPS from Gram-negative bacteria. Endotoxin, released into the bloodstream during microbial infections, causes various pathophysiological effects in mammals, known as bacterial sepsis or systemic inflammatory response syndrome (SIRS). Sepsis is accompanied by the inability to regulate the inflammatory response and can lead to multiorgan failure, shock and often to death [1,2,3,4,5]. Importantly, sepsis is one of the chief causes of death in intensive care units, with mortality rates of ~30%. In Europe, about 0.55 million individuals suffer from sepsis every year, but neither concepts for early diagnosis nor specific therapies have been yet established. Both humoral and cellular defense mechanisms are involved in the inflammatory response. Powerful pro-inflammatory mediators, especially tumor necrosis factor-α (TNF-α), interleukin (IL)-6, IL-1 and IL-8 are produced by endothelial and epithelial cells as well as neutrophils, monocytes, macrophages and lymphocytes at the early onset of sepsis. Liver parenchymal cells are stimulated by TNF-α, IL-1 and IL-6 to produce acute phase proteins. An important acute-phase protein is the glycosylated 58-kDa lipopolysaccharide binding protein (LBP) [6,7] which is upregulated in the context of a signaling pathway at the onset of LPS-induced sepsis [8,9]. In humans, LBP levels reach a maximum on days 2 to 3 during the acute phase of trauma or sepsis [7]. In a previous study [10] LBP serum levels in 180 patients with severe sepsis were analyzed at study entry, after 2 days and at day 7. An increase of LBP levels at day 2 correlated with higher mortality, and serum LBP levels did not differ among patients with Gram-negative, Gram-positive or fungal infections, suggesting that serum LBP levels are of value as a general diagnostic marker of severe sepsis. Early detection of sepsis combined with timely and appropriate interventions are thought to increase the likelihood of survival for patients with sepsis [11].

LBP is member of a family of structurally and functionally related lipid-binding proteins, also including the bactericidal/permeability increasing protein (BPI), phospholipid ester transfer protein (PLTP) and cholesterol ester transfer protein (CLTP) [12,13,14,15,16,17]. LBP binds to the lipid A moiety, the covalently linked lipid component of LPS [18], with reported *K*_d_ values of ~1 (rabbit LBP) and ~60 nM (human LBP) [19,20]. The region of amino acids 91 and 108 of human LBP, enriched in positively charged residues, apparently constitutes the binding site of lipid A and therefore has an essential functional role [21,22]. The C-terminal part of LBP mediates the catalytic transfer of monomeric LPS to membrane-bound CD14 [23,24], resulting in LPS-induced activation of monocytes and macrophages [25,26]. LPS is released from CD14 in the lipid bilayer and binds to a complex of receptors including Toll-like receptor 4 (TLR-4) to initiate intracellular signaling cascades [27] and the transcription of hundreds of genes mediated through nuclear factor κB (NF-κB) [28,29]. A plethora of strategies for the treatment of sepsis has been pursued, especially those that target the mediators of systemic inflammation, but so far without a major breakthrough (for reviews, see references [30,31,32,33]). One problem is the need to personalize treatments for hyperinflamed versus immunoparalyzed patients [33]. Inhibition of LPS-LBP interaction to impede LBP-mediated monomerization of LPS-aggregates or blockage of LPS monomer transfer to CD14 receptors may be promising novel therapeutical strategies in the struggle against septic shock.

SELEX (systematic evolution of ligands by exponential enrichment) is a powerful tool to isolate nucleic acid-based high-affinity ligands (aptamers) for a wide range of molecular targets [34,35]. They usually bind their target structures with high specificity, resulting in the capacity to discriminate closely related target molecules. Potential advantages of aptamers over protein-based molecules include their stability, their robust and simple synthesis, the ease of modification for detection and immobilization purposes, their usually low immunogenicity, and their applicability in cases where antibodies lack sufficient target specificity/affinity or cannot access their target epitopes [36].

Unmodified RNA is highly susceptible to degradation by ribonucleases, a limitation for the use of aptamers in diagnostic or therapeutic applications. However, ribose modifications such as 2′-deoxy, 2′-amino (2′-NH_2_), 2′-fluoro (2′-F), a variety of 2′-*O*-alkyl moieties or locked nucleic acid (LNA) confer resistance to ribonucleases that utilize the 2′-OH group in their reaction mechanism [37]. In vitro selections of nuclease-resistant aptamers were successfully carried out using both 2‘-NH_2_-pyrimidine [38] and 2′-F-pyrimidine ribonucleotides [39].

An in vitro selection, using a 2′-F-pyrimidine RNA library, was performed to isolate nuclease-resistant aptamers that specifically recognize murine LBP (mLBP). Mice are an important animal system for the study of sepsis, and such studies are a prerequisite for potential therapeutic applications in humans. One of three pursued selection strategies led to the successful identification of LBP-specific aptamers with *K*_d_ values in the range of ~200–800 nM. The best binding aptamer (A011) was further characterized by structure probing, boundary experiments and protein binding assays including orthologous human LBP.

## 2. Results and Discussion

### 2.1. In Vitro Selection of LBP-Binding Aptamers

**Figure 1:**
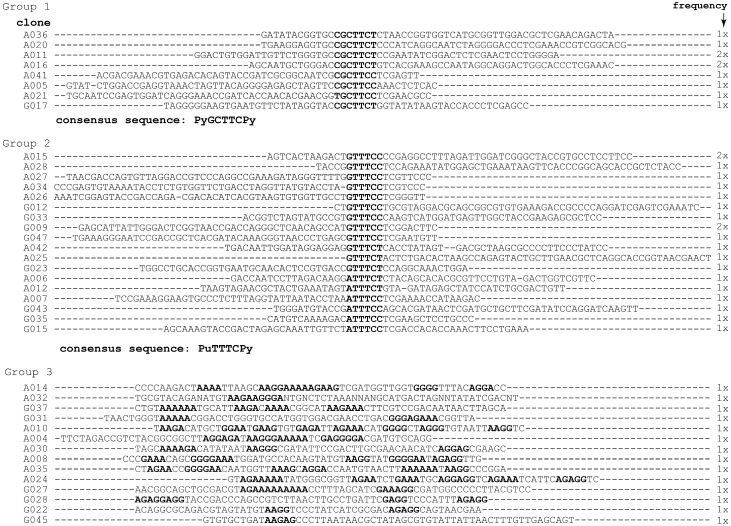
Sequence groups of 2′-F-Y-modified RNA aptamers identified by selection for binding to mLBP. About 100 individual clones were sequenced after eight rounds of selection (see Table 1 and Table 2), and 44 analyzable sequences were divided into three groups based on sequence motifs. For each clone of groups 1 and 2, the sequence of the randomized region is shown and the frequency of occurrence is indicated on the right. Group 1 contained eight different clones with a common sequence motif of seven nt (5′-YGCTTCY), group 2 eighteen clones with a common motif of six nucleotides (5′-RTTTCY). The consensus motifs are highlighted in bold. In the orphan group 3, stretches of four or more consecutive purines are highlighted as well.

We utilized the process of in vitro selection to isolate 2′-F-pyrimidine-modified RNAs (2′-F-Y-RNA) specific for murine LBP (mLBP). A modified RNA pool was synthesized as we had observed beforehand some degradation of unmodified RNA upon incubation with mLBP. Towards this goal, three independent selections were performed, differing in the selection step (binding assay). Initially, nitrocellulose (NC) membranes were used to separate mLBP-aptamer complexes from unbound pool nucleic acids. However, membrane-binding species dominated after several cycles of selection despite involving at least one and up to three preselection steps with NC membranes in the absence of protein. In the second approach, mLBP was immobilized to Ni-NTA magnetic agarose beads as affinity matrix. Here, each selection step (using roughly equimolar amounts of nucleic acid pool and mLBP) was preceded by a preselection step with matrix alone. Pool nucleic acids binding to the matrix alone in the preselection step substantially increased in rounds 6 to 8, reaching 38% in round 8, while the percentage of target binders stagnated (7 ± 1%). The selection was stopped and nucleic acids that bound to mLBP-coated Ni-NTA magnetic agarose beads in round 8 were eluted and subjected to reverse transcription, PCR, plasmid cloning and sequencing. Unfortunately, sequences with motifs of four or more consecutive purines dominated, reminiscent of Ni^2+^-binding motifs identified in previous selections [40]. In parallel, we pursued a third strategy, utilizing primarily Ni-NTA agarose as matrix (see Table 1 for selection parameters). The fraction of matrix binders reached ~20% in rounds 2 to 4, while the fraction of molecules binding to the mLBP-coated matrix did not exceed 2.5% in round 3 and even decreased to below 2% in round 4 (Table 2). Based on our experience of matrix binders prevailing in the two preceding selections (see above), we decided to change the type of matrix for two cycles after round 4 to interrupt the anticipated enrichment of matrix binders. Although chemically related to Ni-NTA agarose, we chose Ni-NTA magnetic agarose beads for round 5 and 6, because it also allowed matrix-coupling of mLBP via its His-tag. Indeed, the fraction of preselection binders decreased and the fraction of target binders increased in rounds 5 and 6. Switching back to Ni-NTA agarose as matrix in round 7 led to an increase of matrix binders, which could be decreased from 31 to 11% in round 8 (Table 2). However, as the fraction of target binders did not significantly increase from round 7 to 8, we stopped the selection at this point. Double-stranded pool DNA from the 8th round was cloned and about 100 clones were sequenced. By using the multiple alignment program Clustal X [41] combined with visual inspection, the different clones could be classified into two groups based on consensus motifs (Figure 1) plus an orphan group 3 with similarities to variants selected with Ni-NTA agarose magnetic beads (see above) and likely representing Ni^2+^-specific binders [40].

Group 1 sequences shared a 7-nt consensus sequence (5′-YGCTTCY), group 2 sequences a 6-nt consensus (5′-RTTTCY). Representatives of each group were tested for binding to mLBP by NC filter-binding. Clones of the first group showed, on average, higher affinity binding than clones of the second, whereas clones of the orphan group did not bind to mLBP.

### 2.2. Secondary Structures of Aptamers

**Figure 2:**
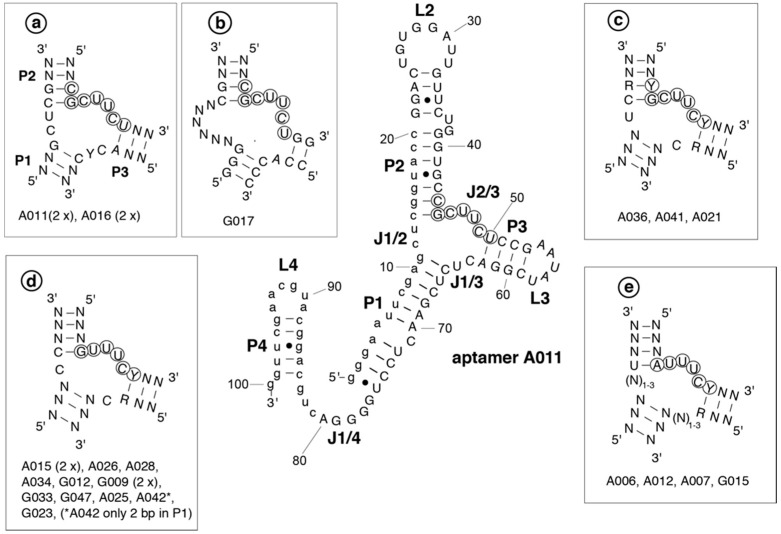
The secondary structure of mLBP-binding aptamer A011, as predicted by mfold (lowest free energy structure) or RNAfold (minimum free energy structure), is shown in the center. Nucleotides of the consensus motif are marked by circles, constant nucleotides of the primer binding sites by small letters. Helical elements were numbered as P1 to P4 (P = paired), loop regions L2 to L4, and joining segments according to the number of the helices they connect (e.g., J1/4). All other aptamers are predicted to adopt the same type of structure with a three helix junction in the core. Differences in the three helix junction were used to define lead structures of types a to e, with individual aptamer clones conforming to the respective subtype indicated below the corresponding structure. Group 1 aptamers constitute structures a to c, group 2 aptamers structures d and e.

The potential secondary structures of selected aptamers were analyzed by Mfold and RNAfold [42,43] and then sorted according to structural similarities in their core regions. Except for A05 and A020 of group 1 and G043 and G035 of group 2, all aptamers adopt a secondary structure similar to that of A011 shown in Figure 2 (center). The common feature is a central three-helix junction, where junction J2/3 exposes the 5′-CUUC and 5′-UUUC elements of the respective consensus motif (group 1: 5′-YGCUUCY; group 2: 5′-RUUUCY; see encircled nucleotides in Figure 2). The aptamer structures not only include the randomized sequence part, but also the constant nucleotides G1 to C20 (small letters in the central A011 structure in Figure 2). The central three-helix junction motif was divided into 5 subgroups primarily based on the consensus motif and the single-stranded nucleotides connecting P1/P2 and P1/P3 (Figure 2, boxed structures a–e). Predicted structures of individual aptamers differed from each other by variations in the three helical arms (helix length, size of apical loops, presence and position of bulges and internal loops). All aptamers analyzed in Figure 2 are predicted to expose four single-stranded pyrimidines of the respective consensus motif in the J2/3 region. This suggests a key role of the four 2′-F-substituted pyrimidines in mLBP binding.

### 2.3. Structural Probing

**Figure 3:**
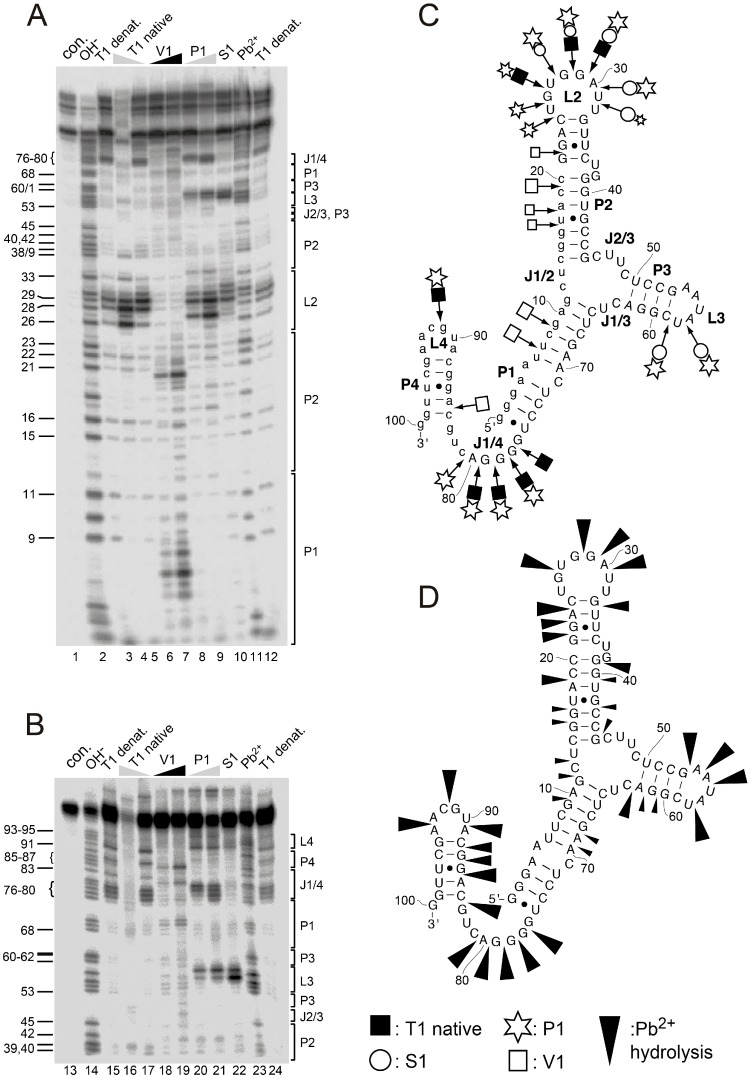
Representative probing experiments using 5′-endlabeled aptamer A011 RNA and analyzed by (**A**) 20% or (**B**) 10% denaturing PAGE. Lanes 1 and 13, undigested RNA control (con.); lanes 2 and 14, limited alkaline hydrolysis; lanes 3, 12, 15 and 24, partial digestion with RNase T1 under denaturing (denat.) conditions; lanes 4, 5, 16 and 17, with two concentrations of RNase T1 under native conditions; lanes 6, 7, 18 and 19, with two concentrations of RNase V1; lanes 8, 9, 20 and 21, with two concentrations of nuclease P1; lane 10 and 22, with nuclease S1; lanes 11 and 23, Pb2+-induced hydrolysis. For experimental details, see Materials and Methods. Alkaline hydrolysis bands and the corresponding structure elements are indicated at the left and right margins, respectively, according to the numbering system presented in Figure 2 (A011, center). (**C**,**D**) Illustration of prominent cleavage sites in the context of the secondary structure of A011, derived from probing experiments such as those shown in panels **A** and **B**. Symbol sizes suggests the relative intensity of cleavage bands based on visual inspection.

Aptamer A011 was further investigated by probing experiments to evaluate its predicted secondary structure. Probing included Pb^2+^-induced hydrolysis, which preferentially affects unstructured single-stranded regions [44]. As single strand-specific enzymatic probes, RNase T1 as well as nucleases S1 and P1 were used. RNase V1 preferentially cleaves helical or structured regions, but also single-stranded regions in stacked conformations [45]. Reaction products derived from Pb^2+^-induced hydrolysis, alkaline hydrolysis and RNase T1 treatment possess 2′,3′-cyclic (or 3′-) phosphates and 5′-hydroxyl termini. In contrast, digestion by nucleases S1, P1 or V1 results in 3′-hydroxyl and 5′-phosphate ends. Due to the replacement of pyrimidines by their 2′-fluoro analogs, sequences were not cleavable at corresponding positions by Pb^2+^ or alkaline hydrolysis (Figure 3A,B). However, nucleases P1 and S1 are capable of cleaving the phosphodiester backbone at the sites of 2′-fluoro modification [46]. A representative probing experiment obtained with 5′-endlabeled aptamer A011 is illustrated in Figure 3A,B, and the most prominent cleavage sites for nucleases V1, P1, S1 and T1 under native conditions have been summarized in the context of the predicted secondary structure (Figure 3C); Pb^2+^-induced hydrolysis sites are summarized in Figure 3D. Nucleases P1, S1 and T1 as well as Pb^2+^ ions primarily cleaved in regions L2, L3 and J1/4, whereas RNase V1 preferentially cleaved in P1, P2 and P4. These findings are consistent with the predicted secondary structure of aptamer A011 (Figure 2), despite the fact that bioinformatic structure predictions were made for RNA and not 2′-F-Y-substituted RNA.

### 2.4. Boundary Experiment

**Figure 4:**
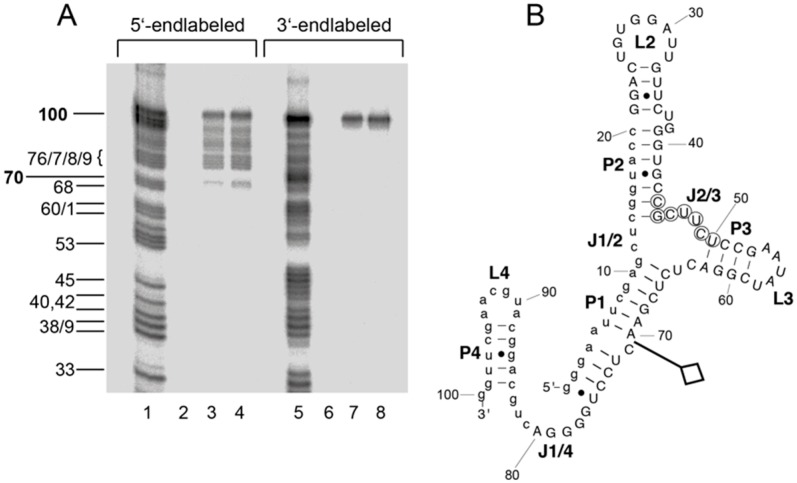
Boundary experiments to derive the minimum structure for efficient mLBP binding. (**A**) Boundary experiments with 5′- or 3′-endlabeled aptamer A011 determined by NC filter binding and 20% denaturing PAGE. Lanes 1 and 5, partial alkaline digest; lanes 3, 4, 7 and 8, intact RNA and alkaline hydrolysis fragments retained on NC filters after incubation for 1 h at 37 °C with 200 nM (lanes 3 and 7) or 400 nM (lanes 4 and 8) mLBP; lanes 2 and 6, as lanes 3, 4, 7 and 8, but omission of mLBP. Alkaline hydrolysis fragments of 5′-endlabeled aptamer A011 are assigned on the left according to the numbering system shown in panel B. (**B**) The 3′ boundary, i.e., the utmost 3′-terminal truncation that has no apparent effect on mLBP binding affinity, is indicated by the flag. With 3‘-endlabeled RNA, the 5′ boundary coincided with the native 5′ end, with possible tolerance for deletion of a few 5′-terminal nucleotides (see panel **A**).

A boundary experiment was performed for aptamer A011 to determine the structural core essential for mLBP binding. 5′- or 3′-endlabeled aptamer was subjected to partial alkaline hydrolysis and then incubated with mLBP. Intact aptamer as well as fragments capable of binding to mLBP were retained with the protein on NC membranes and eluted from membranes in the presence of 7 M urea for analysis by denaturing PAGE (for details, see Materials and Methods). As illustrated in Figure 4, up to 30 nucleotides could be removed from the 3′ end without abrogating binding to mLBP, but truncation of at most a few nucleotides was tolerated at the 5′-end. The result indicated that the 5′-distal part of helix P1 (U6-G11 and C65-A70) is a crucial element of A011 architecture. Thus, a substantial portion of the constant 5′-terminal primer binding sequence is an integral part of the aptamer structure.

### 2.5. Affinity and Selectivity of A011 Binding to mLBP

**Figure 5:**
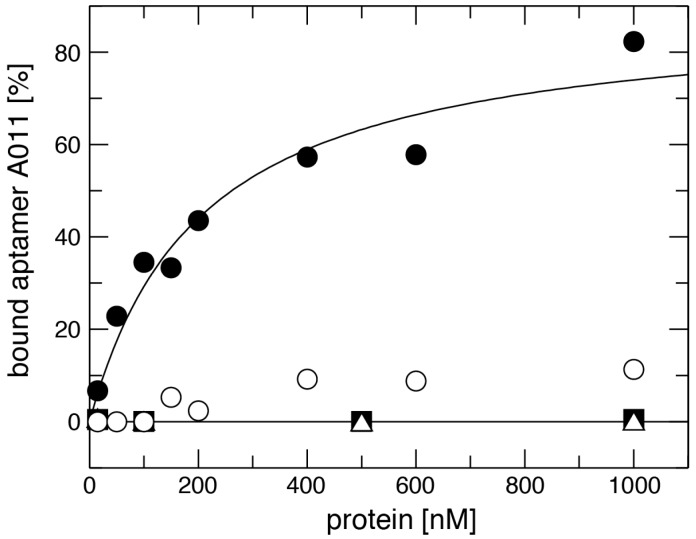
Example experiment illustrating binding of aptamer A011 binding to mLBP (●), hLBP (○), EF-Tu (■) and BSA (∆), determined by NC filter binding assays in the presence of trace amounts of radiolabeled aptamer and increasing excess amounts of the respective protein. For experimental details, see Materials and Methods.

Aptamer affinity for mLBP was investigated by NC filter-binding experiments. A constant low amount (≤5 nM) of radiolabeled aptamer was incubated with increasing concentrations of mLBP (15–1000 nM) and filtered through NC membranes. Screening of the selected aptamers shown in Figure 1 for mLBP binding resulted in *K*_d_ values roughly varying between 200 and 800 nM, with A011 and A036 belonging to the best binders. Comparable affinity of A011 and A036 (both belonging to group 1) correlated with similarity in secondary structure (Figure 2). For A011, a *K*_d_ of 267 ± 48 nM (standard error of the mean) was derived from six individual binding experiments. We included BSA, the bacterial RNA binding protein EF-Tu (from *Thermus thermophilus*) and hLBP to control for the specificity of aptamer A011 binding to mLBP. A representative experiment is shown in Figure 5. BSA and EF-Tu did not result in any binding under these conditions and only very residual binding of A011 to hLBP was observed (Figure 5). Human LBP was a key control, since the protein has 68% sequence homology to murine LBP; hLBP has two additional glycosylation sites (a total of 4), two more than mLBP (UniProt database). The results verify the specificity of A011 for mLBP. A011 therefore expands the repertoire of aptamers addressing sepsis-relevant targets, which also includes LPS-antagonizing ssDNA aptamers that were selected previously [36,47].

## 3. Materials and Methods

### 3.1. Materials

A synthetic pool of DNA molecules containing a 60-nucleotide randomized region and constant 5′- and 3′-terminal sequences (5′-CCA AGC TTG CAT GCC TGC AG [**N**]_60_ GGT ACC GAG CTC GAA TTC CC) was synthesized by NOXXON Pharma (Berlin, Germany). PCR primers were purchased from Metabion (Martinsried, Germany) or IBA GmbH (Göttingen, Germany): primer A (5′-TAATACGACTCACTATA GGG AAT TCG AGC TCG GTA CC; T7 promoter underlined), primer B (5′-CCA AGC TTG CAT GCC TGC AG) and primer C (5′-TAA TAC GAC TCA CTA TAG). The mutant T7 RNA polymerase (Y639F; [48]) was a gift from Jerzy Ciesiolka (Poznan, Poland). Radionucleotides where obtained from Hartmann Analytic (Braunschweig, Germany) or NEN^TM^ Life Science Products, Inc. (Boston, MA, USA), 2′-fluoro-CTP (2′-F-CTP) and 2′-fluoro-UTP (2′-F-UTP) from IBA (Göttingen, Germany), Ni-NTA agarose and magnetic beads from Qiagen (Hilden, Germany). Recombinant murine and human LBP proteins carrying six additional histidine residues at their C-termini (mLBP-His6 and hLBP-His_6_) were from biometec (Greifswald, Germany); for the sake of simplicity, mLBP-His_6_ and hLBP-His_6_ are termed mLPB and hLBP throughout the text. All other reagents and chemicals were of highest purity available and purchased from various commercial sources. *Thermus thermophilus* EF-Tu was a gift from the laboratory of Mathias Sprinzl at Bayreuth University (Bayreuth, Germany) and bovine serum albumin was from Sigma-Aldrich (St. Louis, MO, USA).

### 3.2. RNA Library

Single-stranded (ss) DNA templates were converted to double-stranded (ds) DNA by fill-in polymerization using T7 Sequenase v2.0 (GE Healthcare, formerly Amersham Biosciences, Little Chalfont, UK) after annealing to primer A. Reactions were performed as described previously [49]. For the starting libraries, 2.4 to 2.7 nmol dsDNA template (divided into two samples of 375 µL) was used for in vitro transcription with T7 RNA polymerase Y639F under the following conditions: 40 mM Tris/HCl pH 8.0, 20 mM MgCl_2_, 1 mM spermidine, 0.01% Triton^®^ X-100, 5 mM DTT, 1.25 mM each 2′-F-CTP and 2′-F-UTP, 1.25 mM each ATP and GTP, 1.11 MBq [α-^32^P]-ATP, 93 ng/µL T7 RNA polymerase. Incubation was performed at 37 °C for 3 to 4 h. In subsequent selection rounds, T7 transcription was performed with 0.15 to 0.5 nmol DNA template in a final volume of 150 to 190 µL.

### 3.3. In Vitro Selection

The selection matrix was prepared as follows: 80 µL Ni-NTA agarose suspension were washed twice in 400 µL ddH_2_O and twice in 400 µL binding buffer (140 mM NaCl, 2.7 mM KCl, 10 mM Na_2_HPO_4_, 1.76 mM KH_2_PO_4_, 2 mM MgCl_2_), at each step separating matrix and supernatant by centrifugation for 10 s at ~10,000× *g*. After the last washing step, the agarose was resuspended in 360 µL binding buffer and combined with 40 µL (40 µg) of mLBP-His_6_. After incubation for 45 min at room temperature with occasional gentle shaking, the sample was centrifuged for 5 min at 1000× *g*. The supernatant containing unbound protein was removed and the agarose matrix was resuspended in 400 µL binding buffer and centrifuged again. This wash step was repeated once, and the Ni-NTA-agarose, coated with mLBP, was then resuspended in 160 µL binding buffer.

Radiolabeled RNAs were purified by denaturing PAGE, gel elution and ethanol precipitation [50]. The 2′-F-Y-modified starting RNA pool (550 pmol, ~3.3 × 10^14^ molecules) was dissolved in binding buffer (see above), adjusting the concentration to 2.9 µM (187 µL), followed by heating to 90 °C for 4 min and cooling to room temperature. This refolded RNA pool was first applied to a 1-mL preselection column containing 50 µL Ni-NTA agarose without mLBP and equilibrated with binding buffer, followed by incubation for 20 min at 37 °C. The preselection column was drained by short centrifugation in a microcentrifuge. The flowthrough RNA fraction from the preselection column was used as the starting pool for the selection.

The flowthrough from the preselection column was combined with the mLBP-coated Ni-NTA-agarose (see above) in a final volume of 400 µL binding buffer, followed by incubation for 1 h at 37 °C with occasional shaking. After two washes (see above) with 200 µL binding buffer to remove non-binding or weakly binding RNAs, bound RNA was eluted by incubation with 200 µL of elution buffer (140 mM NaCl, 2.7 mM KCl, 10 mM Na_2_HPO_4_, 1.76 mM KH_2_PO_4_, 2 mM MgCl_2_, 300 mM imidazole, 7 M urea) for 1 h at 37 °C with occasional shaking. The eluate was extracted with phenol/chloroform (1:1) and chloroform and RNA was precipitated with ethanol in the presence of 20 µg glycogen as carrier. RNA was reverse-transcribed to DNA, amplified by PCR after sequenase fill-in polymerization (see above), and finally transcribed to RNA for the next round of selection. Reverse transcription was performed as described [49], except that incubation was for 1 h at 42 °C.

In total, eight rounds of in vitro selection were performed. Compared with the first round, mLBP-His_6_ was coupled to a smaller volume of Ni-NTA agarose suspension (20 µL in round 2–4, 7 and 8) and the 2′-F-Y-RNA pools were dissolved in ~100 µL before the preselection step (for pool and mLBP concentrations, see Table 1). In rounds 5 and 6, the selection step was performed with mLBP-His_6_ coupled to Ni-NTA agarose magnetic beads (~30 µL). Here, separation of supernatant and beads was achieved with a magnet, not by centrifugation. The selection step in rounds 2 to 8 was carried out in a volume of 100–300 µL (Table 1), washing and elution were performed as described for round 1 above. After round eight, PCR products were cloned into the vector provided with the TOPO TA Cloning^®^ Kit (Thermo Fisher Scientific Invitrogen, Waltham, MA, USA) according to the supplier’s instructions. About 100 individual clones were sequenced.

### 3.4. 3′- and 5′-Endlabeling

3′- and 5′-endlabeling of aptamers was performed as described [51], using T4 RNA ligase and T4 polynucleotide kinase [51]; 5′-terminal dephosphorylation was conducted with calf intestine alkaline phosphatase according to the manufacturer’s protocol (all three enzymes from Thermo Fisher Scientific, Waltham, MA, USA). Labelled RNAs were purified by denaturing PAGE, bands were visualized by phosphorimaging using a BAS-1000 Bio-Imaging Analyzer (Fujifilm, Tokyo, Japan), excised and eluted in 1 M NaOAc, pH 4.6. RNA was precipitated with ethanol in the presence of 20 µg glycogen.

### 3.5. Determination of Equilibrium Dissociation Constants

Equilibrium dissociation constants (*K*_d_) of aptamers for mLBP were determined by nitrocellulose (NC) filter-binding assays. RNAs were internally labeled with [α-^32^P]-ATP during in vitro transcription or post-transcriptionally at their 5′-ends with [γ-^32^P]-ATP (see above). Labeled RNA aptamers (≤0.5 pmol) were denatured and refolded in 90 µL of binding buffer as described for the in vitro selection process, and then incubated with increasing concentrations of mLBP-His_6_ (15 nM to 1 µM) for 1 h at 37 °C (final volume 100 µL). Mixtures were filtered through an NC filter (25 mm Millipore, HAWP, 0.45 µm) pre-wetted with binding buffer, and washed with 0.3 mL binding buffer. Filters were air-dried on Whatman filter paper (Sigma-Aldrich, St. Louis, MO, USA). Bound RNAs were detected by phosphorimaging and quantified using the analysis software PCBAS v2.09 (Raytest, Straubenhardt, Germany). The percentage of RNA bound to filters in the absence of mLBP was subtracted from all data points. The *K*_d_ value for aptamer A011 was derived from six independent experiments.

### 3.6. Enzymatic and Chemical Structure Probing

Before chemical or enzymatic probing, endlabeled aptamer A011 was denatured and refolded in binding buffer (140 mM NaCl, 2.7 mM KCl, 10 mM Na_2_HPO_4_, 1.76 mM KH_2_PO_4_, 2 mM MgCl_2_) as described above. All reactions with A011 (40,000–150,000 Cherenkov cpm per reaction) were done in the presence of 1.5 µg (Pb^2+^-hydrolysis) or 2.5 µg (all other reactions) of carrier tRNA (yeast tRNA, Sigma-Aldrich, St. Louis, MO, USA). Alkaline digestion was performed in 50 mM NaOH and 0.5 mM EDTA for 90 s at 110 °C in a thermoblock. Enzymatic digestions were performed as follows: RNase T1 (Thermo Fisher Scientific, Waltham, MA, USA) either in 20 mM sodium citrat (pH 5.0), 7 mM urea, 1 mM EDTA and 1 unit RNase T1 for 30 min at 55 °C (denaturing conditions) or in binding buffer (see above) with 0.5 or 0.05 units for 20 min at room temperature (RT); 0.01 or 0.05 units RNase V1 (Thermo Fisher Scientific Pierce) in binding buffer for 20 min at RT; 1 unit RNase S1 (Thermo Fisher Scientific, Waltham, MA, USA) in binding buffer containing 4.5 mM ZnSO_4_ for 20 min at RT; 0.01 or 0.05 units nuclease P1 (Sigma-Aldrich, St. Louis, MO, USA) in binding buffer containing 0.4 mM ZnSO_4_ for 20 min at RT. Pb^2+^-hydrolysis was performed in 50 mM Tris/HCl (pH 7.0), 100 mM Mg(OAc)_2_ and 50 mM NH_4_OAc for 10 min at 37 °C. Hydrolysis reactions were started by adding 1 µL of freshly dissolved lead acetate solution (100 mM) to 4 µL of RNA solution. Hydrolysis was stopped by addition of 7 µL denaturing loading buffer (2.3 M urea, 1 × TBE, 66% (*v*/*v*) formamide, 0.05% (*w*/*v*) each bromophenol blue and xylene cyanol) containing 120 mM EDTA, followed by shock-freezing in liquid nitrogen. All probing experiments were analyzed by 10% and 20% denaturing PAGE.

### 3.7. Boundary Experiments

Trace amounts of 5′- or 3′-endlabeled aptamer A011, that is (2–2.5) × 10^5^ Cherenkov cpm, were mixed with 2.5 µg yeast tRNA (Sigma-Aldrich, St. Louis, MO, USA) and partially hydrolyzed under alkaline conditions (50 mM NaHCO_3_) for 10 min at 90 °C (final volume 12 µL). Samples were concentrated by ethanol precipitation and redissolved in binding buffer, denatured and refolded (see above), followed by incubation with mLBP (200 nM or 400 nM) for 1 h at 37 °C (final volume 100 µL). Mixtures were passed through an NC filter (25 mm Millipore, HAWP, 0.45 µm), pre-wetted with binding buffer and washed with 0.3 mL binding buffer. Bound RNA fragments were eluted from filters with 200 µL 7 M urea, extracted with phenol/chloroform and chloroform, followed by ethanol precipitation in the presence of 20 µg glycogen. Fragments were analyzed by denaturing PAGE along with the starting hydrolysate before the addition of mLBP.

## 4. Future Prospects

The present length of A011 (100 nt) is suboptimal for its production by chemical synthesis in terms of costs and yields. So far, deletion of nt 76–100 resulted in a fivefold reduction of mLBP affinity. Introduction of LNA modifications into the helical parts of A011 by may lead to gains in affinity by rigidifying its folded structure, which may in turn allow some shortening of helices without considerable losses in mLBP affinity. The group 1 and 2 consensus motifs of our mLBP aptamers mainly consist of 2′-F-pyrimidines. Future protection and modification experiments will have to show if the consensus motifs are indeed major recognition epitopes and, if yes, to which extent the 2′-F-ribose moieties could be replaced with RNA or DNA building blocks or other analogs without compromising affinity and specificity. For improvement of aptamer affinity, a reselection using a partly randomized library based on the initial aptamer sequence may be considered. Eventually, future experiments are awaited to demonstrate that such aptamers are able to inhibit LPS-induced signal cascades in murine cells. This will be crucial to assess whether the affinity of aptamer A011 for mLBP (*K*_d_ ~250 nM) is sufficiently high to elicit the desired biological effects. Finally, aptamer A011 was shown to be highly specific for murine LBP. As a consequence, aptamers specific for human LBP have to be selected for diagnostic or therapeutic applications in human health care. Our successful selection of mLBP-specific aptamers makes it likely that the related hLBP will be a suitable target as well, and future studies targeting hLBP will benefit from the knowledge gained in this study. Additionally, other matrix:protein coupling chemistries or counter selection with other proteins may improve the selection outcome; for example, counter selection with mLBP when using hLBP as target (or vice versa) may allow selection of aptamers binding to the targeted LBP with enhanced affinity and specificity.

## Figures and Tables

**Table 1:** Selection parameters and elution results of individual selection rounds. The concentration of the 2′-F-Y-RNA pool (2nd column) is that before preselection. The selection was performed with Ni-NTA agarose in rounds 1-4, 7 and 8, and with Ni-NTA agarose magnetic beads in rounds 5 and 6 (indicated by the subscript “MB”).

Round	2′-F-Y-RNA (μM)	mLBP (μM)	Total Volume (μl)	RNA:mLBP ratio	Preselections
1	1.37	1.6	400	1.0 : 1.2	+
2	0.85	0.9	300	1.0 : 1.3	+
3	0.45	0.8	200	1.0 : 1.8	+
4	0.5	0.8	200	1.0 : 1.5	+
5_MB_	0.48	0.8	100	1.0 : 1.6	+
6_MB_	0.7	0.8	200	1.0 : 1.1	+
7	0.79	0.8	200	1.0 : 1.0	+
8	1.1	0.8	200	1.4 : 1.0	+

**Table 2:** The fractions (in %) of the 2′-F-pyrimidine (Y)-modified RNA pool that remained bound to the matrix in the preselection step (central column) and that eluted after incubation with matrix-bound mLBP in each of the eight selection rounds (right column).

Ni-NTA Agarose Matrix		
Round	2′-F-Y-RNA Preselection (%)	2′-F-Y-RNA Selection (%)
1	10.70	1.28
2	17.00	0.96
3	21.00	2.50
4	18.60	1.83
5_MB_	8.00	4.10
6_MB_	7.53	4.92
7	31.00	2.64
8	11.00	2.72

Note that Ni-NTA magnetic agarose (MB) instead of Ni-NTA agarose beads were used in rounds 5 and 6.
